# Motivation Levels and Goals for the Practice of Physical Exercise in Five Different Modalities: A Correspondence Analysis

**DOI:** 10.3389/fpsyg.2021.793238

**Published:** 2021-12-21

**Authors:** Juliana Correia Borges, Gilson Gonçalves de Oliveira Filho, Claudio Andre Barbosa de Lira, Ronaldo Angelo Dias da Silva, Eduardo da Silva Alves, Mateus Joacir Benvenutti, João Paulo Pereira Rosa

**Affiliations:** ^1^Curso de Educação Física - Prado, Centro Universitário Estácio, Belo Horizonte, Brazil; ^2^Faculdade de Educação Física e Dança, Universidade Federal de Goiás, Goiânia, Brazil; ^3^Departamento de Ciências da Saúde, Universidade Estadual de Santa Cruz, Ilhéus, Brazil; ^4^Programa de Pós-graduação em Biociências e Fisiopatologia, Universidade Estadual de Maringá, Maringá, Brazil

**Keywords:** exercise motivation, adherence, health promotion, motivation level, lifestyle

## Abstract

The identification of the practitioner’s profile regarding their motivation level for physical exercise engagement could be a behavioral strategy to increase exercise adherence. The present study investigates the associations between motivation levels, modalities practiced, and goals concerning the practice of physical exercise among physical exercise practitioners. A total of 100 physical exercise practitioners, of which 67 were women, took part in this study. The participants were engaged in extreme fitness program, strength training, fight training, Pilates, and functional training. Motivation level (BREQ-3) and expectations regarding regular physical exercise (IMPRAF-54) were assessed. A multiple correspondence analysis demonstrates preferential relationships between descriptive and non-inferential variables. Strength training and fight training practitioners seek these modalities with the goals of “Health” and “Aesthetics,” demonstrating low autonomy in relation to the behavior for the practice of physical exercise. Extreme conditioning program and functional training practitioners have as goal “Pleasure,” demonstrating medium and high levels of autonomy for such practice and Pilates practitioners have the goal of “Stress Control.” To promote and encourage the regular practice of physical exercise, this strategy could be used to take actions that increase the public’s intention to start or continue in a physical exercise program.

## Introduction

Regular physical exercise has been shown to be an effective way to promote health and increasing and/or improving physical and psychological status of practitioners (Mandolesi et al., 2018). However, the prevalence of individuals who do not engage in a regular physical exercise program is still high (Middelkamp et al., 2017; Martins et al., 2021). In this context, it has been investigated how human behavior is associated with physical exercise practice (Sallis, 1999) with some determinants of this behavior being identified as a crucial on intention toward exercise participation (Rodrigues et al., 2018). Low level of motivation, self-efficacy, little social or cultural support, and shortage of time are considered the main reasons for individuals to start but not to adhere to a regular physical exercise program (Sherwood and Jeffery, 2000). Additionally, more recent studies used a multi-section survey of motivational, emotional, and cognitive-related variables to test if past behavior has affected exercise-related outcomes (i.e., adherence) for gym clients according to their previous exercise experience (Rodrigues et al., 2021).

The result of exercise prescription is a consequence of periodization techniques associated with the observation of behavioral and environmental elements; thus, the need to observe the psychobiological factors that can modulate adherence to physical exercise are of crucial relevance (Lucini and Pagani, 2021). Based on this, some theories have been proposed that it is better to understand human motivations for engaging in exercise activities. One of the most current theories on motivation is the self-determination theory (SDT), presented by Deci and Ryan (1985). This theory explains that through different levels of motivation, ranging from amotivation, extrinsic motivation, and intrinsic motivation, the individual is more likely to initiate and maintain a behavioral change. For instance, participants’ awareness of the importance of physical training for the purposes of improving and maintaining health and quality of life is a relevant aspect for maintaining motivation in an exercise program. Coherent with the SDT, Vallerand (1997) developed the hierarchical model of evidencing the influence of social factors on the three basic psychological needs: autonomy (i.e., personal choice), competence (i.e., confident feedback), and relatedness (i.e., social interactions). The basic psychological needs triad leads to more autonomous motivation forms such as enjoyment, well-being, and positive affect as major contributors to exercise adherence (Ryan and Deci, 2017).

A plethora of reasons may be considered when the topic is “motivation to exercise.” Some individuals show a preference for performing predominantly individual modalities (such as running or resistance training), whereas others prefer group activities (such as dance and martial arts; Box et al., 2019). Some gym members were motivated by factors such as positive health and physical fitness at the beginning of fitness journey (first year of fitness club membership), while higher levels of the motives enjoyment and challenge were associated with regular exercise by more experienced practitioners (Gjestvang et al., 2020).

Besides that, identifying preferences prior to starting a physical exercise program could provide practitioners with higher motivational level to adopt physical exercise in their daily routines and to also direct them to a sort of exercise according to their preferences. For example, the association of physical activity with social practices may set subjects in a physical activity spot that meets their needs and goals, providing better results of adherence to a physical exercise program (Rosa et al., 2015).

Based on previous studies about the motivation for engaging in different physical activities showing differences in motives for sports and exercises (Hsu and Valentova, 2020), there are differences by sex and age among different physical activities, in a way that fitness/health motivation increased with age, while appearance motivation decreased, as well as regarding sex, women reported higher interest/enjoyment than men (Hsu and Valentova, 2020).

Consequently, identifying the practitioner’s profile regarding their level of motivation and goals with the physical exercise practice would help professionals in indicating the modalities and in prescribing exercise protocols that are aligned with the subject’s preferences. This is thought to be a form of behavioral strategy that could increase the rates of initiation and maintenance of regular physical exercise.

Thus, the present study investigates the associations between motivation levels (low, medium, and high levels of autonomy), modalities practiced (extreme fitness program, strength training, fight training, Pilates, and functional training), and goals (sociability, competitiveness, stress control, health, aesthetics, and pleasure) concerning the practice of physical exercise.

## Materials and Methods

### Participants

A non-probabilistic sampling (convenience) was used. Men and women, aged between 18 and 40 years, practitioners of extreme fitness program, strength training, fight training, Pilates, and functional training participated in the study. Only participants who had been enrolled in the before mentioned physical exercise modalities for at least 1 month were included. Participants who practiced any of the modalities for more than 12 months were not included in the study. This study was approved to the Research Ethics Committee of the Universidade Estácio de Sá (CAAE 34538220.2.0000.5284). All participants were informed about the procedures, discomforts, and risks involved in the assessment processes.

### Design and Procedure

With prior authorization granted by the business managers from where participants attended to practice the exercise classes (Belo Horizonte, Minas Gerais, Brazil), a total of 100 participants (33 men and 67 women) took part in this study. The practitioners had an average age of 29.4 ± 6.7 years, body mass of 71.0 ± 17.0 kg, height of 1.67 ± 0.08 m, and body mass index (BMI) of 25.2 ± 4.4 kg/m^2^.

Participants answered the following questionnaires: The Inventory of Motivation for the Regular Practice of Physical Activity and/or Sports (IMPRAF-54; Barbosa, 2006) to assess the motivational dimensions associated with the regular exercise practice and the Behavioral Regulation in Exercise Questionnaire (BREQ-3 Portuguese version; Guedes and Sofiati, 2015) to characterize the motivational profile (intrinsic and extrinsic motivation) anchored in the SDT (Deci and Ryan, 2012).

The IMPRAF-54 comprises 54 items, each of which has five-point Likert scales that ranged from 1 to 5. A score of 1 meant “this motivates me very little,” and 5 meant, “this motivates me a great deal.” They were clustered according to the particular dimension of motivation that is being assessed: stress control (e.g., releasing mental tension), health (e.g., maintaining physical fitness), sociability (e.g., spending time with friends), competitiveness (e.g., winning a competition), aesthetic (e.g., having an attractive appearance), and pleasure (e.g., personal enjoyment). Each dimension was assessed using the same number of questions.

The BREQ-3 inventory comprises 19 Likert items, each of which has five possible answers being scored on a scale from 0 to 4 (0 = Not true for me; 4 = Very true for me). The questionnaire assesses five constructs based on the five types of motivation in the SDT: amotivation (e.g., “I think that exercising is a waste of time”), external regulation (e.g., “I exercise because other people tell me I should”), introjected regulation (e.g., “I feel guilty when I do not exercise”), identified regulation (e.g., “I value the benefits/advantages of exercising”), and intrinsic motivation (e.g., “I enjoy my exercise sessions”). The relative autonomy index was used to gain insight into the degree of autonomy, since the five types of motivation are located in the continuum of self-determination.

Prior to the pandemic period COVID-19, questionnaires were distributed to 24 participants and standardized as an interview, being conducted in individual environments to preserve participant’s privacy. Due to the pandemic, however, the remaining participants answered the questionnaires remotely *via* Google Forms, with the researchers inspecting the filling of the forms remotely to clarify any doubts. Participants received the consent form by email through a link from assinaturagratis.com—by Contraktor—and the researchers’ received confirmation of the consent term’s signature.

### Statistical Analysis

Data normality was assessed using the Kolmogorov–Smirnov test and non-normal data were observed for all variables. Therefore, after the removal of the means of continuous variables, standardization by *z*-score was performed. The analysis of variance of the mean scores of the groups (ANOVA one-way) was used to identify differences between the modalities (extreme fitness program, strength training, fight training, Pilates, and functional training) regarding the motivation scores (BREQ-3, IMPRAF-54, and relative autonomy index). When differences were identified, the Bonferroni *post hoc* test was used. For continuous variables, the results are expressed as mean ± standard deviation. In all statistical procedures, the level of significance adopted was *p* < 0.05. SPSS Statistics software (v21, IBM, United States) was used for statistical analysis.

A multiple correspondence analysis was performed to group the participants according to the pattern of their responses to the study variables. This analysis allows us to demonstrate preferential relationships between descriptive (categorical) and non-inferential variables. Thus, we created profiles for the participants according to the motivation self-determination index (low, medium, and high levels of autonomy), practiced modalities (extreme fitness program, strength training, fight training, Pilates, and functional training), and objectives of physical exercise practice in five of the six dimensions obtained from the IMPRAF-54 questionnaire (competitiveness, stress control, health, aesthetics, and pleasure). The “Eigenvalue” was used as the model’s quality index, which shows the extent to which each variable contributed to the definition of the profile studied. Cronbach’s alpha value was used as a measure of internal consistency, presenting the mean of inter-correlation between items.

## Results

Table 1 shows sample characteristics of this study. There was an age difference between participants of the extreme fitness program and the strength training (*p* = 0.02) and between participants of the extreme fitness program and fight training (*p* < 0.01).

**Table 1:** Study sample characteristics.

	Modalidade					ANOVA one-way	
	Pilates (*n* = 20)	Extreme fitness program (*n* = 20)	Strength training (*n* = 20)	Fight training (*n* = 20)	Functional training (*n* = 20)	*F*	*p*
Age (years)	29.2 ± 5.2	33.9 ± 5.4	27.9 ± 6.2	25.1 ± 6.8	30.8 ± 6.8	5.67	<0.01^*^
Weight (kg)	75.0 ± 21.6	67.9 ± 15.4	66.3 ± 9.9	72.4 ± 17.5	73.1 ± 17.9	0.93	0.44
Height (m)	1.68 ± 0.08	1.64 ± 0.05	1.66 ± 0.08	1.70 ± 0.08	1.65 ± 0.08	2.11	0.08
BMI (kg/m^2^)	25.9 ± 5.4	25.1 ± 4.6	23.8 ± 2.3	24.7 ± 4.5	26.5 ± 4.4	1.08	0.37

Data presented as mean ± standard deviation. BMI, body mass index.

* Statistical difference between extreme fitness program and strength training (*p* = 0.02) and extreme fitness program and fight training (*p* < 0.01).

Table 2 shows the values of motivational characteristics and objectives for the participants’ exercise practice. No significant differences between modalities were found concerning the levels of motivation measured by the BREQ-3 questionnaire. Regarding the IMPRAF-54, there was a difference between the modalities for the dimensions: “sociability” (*p* = 0.01), “competitiveness” (*p* < 0.01), and “aesthetics” (*p* = 0.01). Bonferroni’s *post hoc* revealed significant difference in “sociability” between fight training and extreme conditioning program (*p* < 0.01), a difference in “competitiveness” between fight training and the extreme conditioning program (*p* = 0.05), strength training (*p* = 0.02), and Pilates (*p* = 0.01), and a difference in “aesthetics” between strength training and the extreme conditioning program (*p* < 0.01).

**Table 2:** Motivational characteristics of participants.

Questionnaire	Modalidade					ANOVA one-way	
	Pilates	Extreme fitness program	Strength training	Fight training	Functional training	*F*	*p*
*BREQ-3*							
Amotivation	0.20 ± 0.43	0.53 ± 1.13	0.26 ± 0.54	0.16 ± 0.39	0.45 ± 0.83	1.18	0.31
External regulation	1.20 ± 1.05	1.05 ± 0.94	0.90 ± 1.20	1.00 ± 0.85	1.50 ± 1.05	1.36	0.26
Introjected regulation	2.80 ± 0.69	2.55 ± 1.09	3.00 ± 0.85	2.65 ± 0.87	2.70 ± 1.03	2.57	0.09
Identified regulation	3.62 ± 0.40	3.35 ± 0.57	3.35 ± 0.76	3.46 ± 0.52	3.38 ± 0.70	1.27	0.29
Integrated regulation	2.41 ± 0.48	2.10 ± 0.70	2.51 ± 0.72	2.48 ± 0.67	2.37 ± 0.70	2.14	0.13
Intrinsic regulation	3.63 ± 0.40	3.37 ± 0.63	3.50 ± 0.63	3.58 ± 0.50	3.40 ± 0.76	1.40	0.25
RAI	13.56 ± 3.58	11.41 ± 4.78	13.29 ± 6.23	14.06 ± 3.27	11.28 ± 5.79	1.61	0.21
*IMPRAF-54*							
Stress control	32.30 ± 7.23	33.00 ± 6.11	30.55 ± 8.03	31.35 ± 5.95	32.50 ± 6.80	0.40	0.80
Health	37.20 ± 2.64	35.50 ± 3.56	36.00 ± 5.24	35.50 ± 4.19	35.50 ± 4.90	0.59	0.66
Sociability	26.25 ± 7.74	19.25 ± 9.16	24.15 ± 9.66	29.65 ± 8.98	24.45 ± 11.62	3.14	0.01^*^
Competitiveness	16.70 ± 9.19	11.35 ± 5.81	15.80 ± 7.08	24.80 ± 10.06	19.05 ± 11.83	5.92	<0.01^*^
Aesthetics	34.15 ± 5.18	28.05 ± 10.07	36.55 ± 3.69	32.10 ± 8.15	32.30 ± 8.66	3.55	0.01^*^
Pleasure	37.75 ± 2.42	35.40 ± 5.43	36.85 ± 5.06	37.05 ± 3.54	37.05 ± 3.66	0.85	0.49

Data presented as mean ± standard deviation. RAI, relative autonomy index; BREQ-3, Behavioral Regulation in Exercise Questionnaire; IMPRAF-54, Motivation Inventory for Regular Physical Activity Practice.

* Statistical differences between the fight training and the extreme fitness program for the “Sociability” dimension (*p* < 0.01), difference between the fight training for the extreme fitness program (*p* = 0.05), strength training (*p* = 0.02), and Pilates (*p* = 0.01) for the “competitiveness” dimension and differences between the strength training and the extreme fitness program (*p* < 0.01) for the “Aesthetics” dimension of the IMPRAF-54.

**Figure 1:**
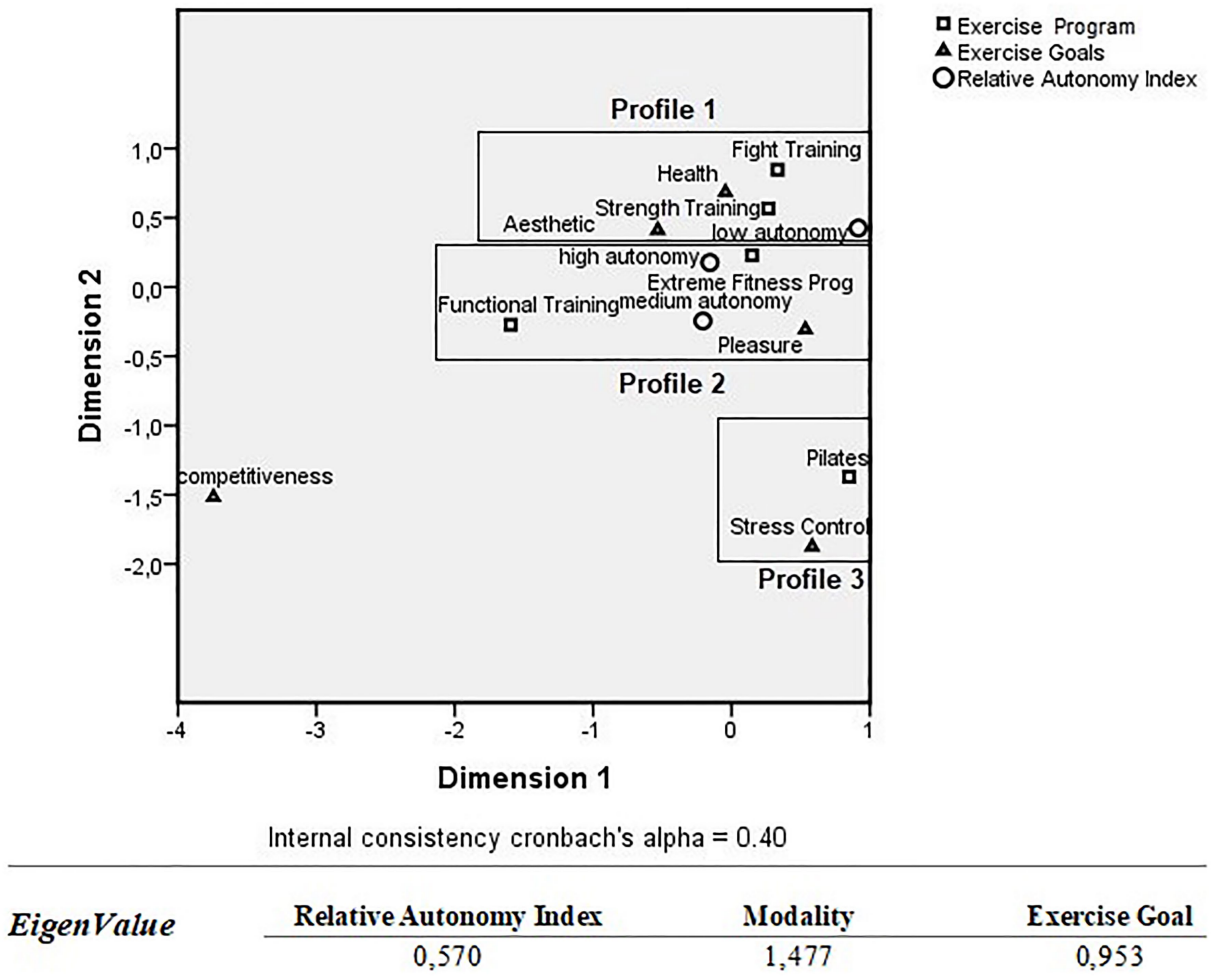
Perceptual mapping between exercise goals, modalities and levels of self-determination of behavior regarding physical exercise practice.

Figure 1 shows the profiles observed when associating modality, objective, and self-determination index in relation to the practice of physical exercise. Three profiles were created with scale values for each modality, objective, and level of autonomy for the practice of physical exercise, being plotted in the form of a perceptual map. This figure allows a view of the interrelationships between the variables of interest, with the grouping in the upper right corner showing that the participants of the strength training and fight training modalities seek the modalities with the objective of “health” and “aesthetics,” demonstrating low autonomy behavior in relation to the practice of physical exercise (Profile 1). Also, in the right corner of the map, the grouping of the extreme conditioning program and functional training modalities can be seen with the practitioners aiming to seek for “pleasure” with the practice of physical exercise, demonstrating medium and high levels of autonomy for such practice (Profile 2).

Also, it was observed in the lower right corner that the participants of the Pilates modality have the objective of “stress control.” The variable “competitiveness” appears in the model. However, it was not a discriminator between the grouped variables as well as between the observed profiles. When evaluating the Eigenvalue, it was verified which measures provided a relative size (importance) of this dimension to the perceptual map. It was also verified that the category “modality” was more important for the map (1.47), followed by the variables “objective of physical exercise” (0.95) and “self-determination index” (0.57). Cronbach’s alpha value was used as a measure of internal consistency, presenting the mean of inter-correlation between items. The present model had an internal consistency of 0.40 (reasonable), according to Landis and Koch (1977).

## Discussion

From an exploratory and visual viewpoints, this study aimed to demonstrate the associations between different motivational factors, personal characteristics regarding the objective for the practice, and the modalities of physical exercise, so that profiles can be considered in the prescription of physical training and provide greater adherence to regular physical exercise.

In the perceptual map shown in Figure 1, there are some associations between motivational factors and physical exercise modalities, from which three profiles can be determined. By outlining a first profile (profile 1), it was verified that the participants of the strength training and fight training modalities seek these kinds of activities with the objective of health and aesthetics and with low autonomy, suggesting a less self-determined motivation profile (Sproule et al., 2007).

There are many physiological and psychological benefits of regular physical exercise. Among the various motivations for adopting this behavior, there is the search for improving body appearance. For example, increasing muscle mass and losing or controlling body mass are among the most documented in the scientific literature (Hausenblas and Fallon, 2006). Such a search for an “ideal physique” is highly encouraged in the contemporary society. Several manufacturers of fitness products and programs exclusively focus on potential improvements in appearance and body composition, utilize television commercials, and Internet advertisements that promote new products and methods with the results guaranteed for an ideal physique end (Anderson et al., 2004). However, such social pressures (e.g., media, friends, and family) for a “perfect” body can facilitate the development of body image disorders, since some physical standards are difficult to achieve with diet and exercise alone (Thompson et al., 1999). Thus, negative body image is a driving ingredient for a variety of health problems, such as depression, obesity, body dysmorphic disorder, and some eating disorders (Stice, 2002).

On the other hand, there is an increase in the general public’s understanding of the benefits of physical exercise in addition to aesthetic factors. In the past, strength training was generally practiced by a specific niche of individuals: Men involved in sports such as weightlifting and bodybuilding, and, therefore, individuals who did not align with such goals do not engage in strength training (Westcott, 2012). With recent scientific advancements, however, it has been observed that strength training benefits all its practitioners, as it is effective in promoting physical gains, including physical performance, movement control, gait speed, and functional independence (Westcott, 2012). Strength training also helps in the prevention and control of non-communicable chronic diseases such as type-2 diabetes, hypertension, dyslipidemia, and osteoporosis, among many others (Westcott, 2012).

In addition to these physical benefits, studies have shown that strength training can also help psychologically. Gordon et al. (2020) showed that regular strength training substantially reduces anxiety in healthy young adults. Other evidence demonstrated that strength training reduces symptoms of depression and other mental disorders, increases feelings of happiness and contentment in adults (Gordon et al., 2018). Fighting, martial arts, and combat modalities are traditionally practiced to minimize stress. There is a growing body of evidence on the long-term effects of martial arts practice, with most of these studies pointing to positive psychosocial outcomes (Binder, 2007). Based on these results, it is possible to infer that practitioners of these modalities recognize and appreciate the results and benefits of the modalities in a more positive and meaningful way, either by an extrinsically motivated behavior of valuing the activity (identified extrinsic motivation) or by satisfaction and pleasure which physical exercise itself provides (intrinsic motivation).

In the present study, participants in the strength training and fight training modalities had low autonomy. According to the SDT, a subject is autonomous when they act according to their authentic interests or integrated values and desires (Deci and Ryan, 2012). Therefore, participants in these modalities presented a controlled behavior, in which actions are determined by behaving in specific ways and/or by external reasons regardless of their values or interests (Chirkov et al., 2003).

It is visually evident in the perceptual map (Figure 1) that a second group (Profile 2) represented by participants with a motivation profile focused on the practice of the extreme conditioning and the functional training program, with the objective of predominantly obtaining pleasure. The intrinsic pleasure in the practice of physical exercise is characterized by individuals who are motivated to engage in activities in which they experience interest, feel competent, and perceive the causal locus for the practice (Reiss, 2004).

The results of the present study corroborate the findings of the review by Dominski et al. (2020) which evaluated the motivational characteristics of individuals who engage in the extreme conditioning program training modality. By evaluating 14 studies, the review reported that, compared to other forms of training, the motivational characteristics of individuals involved in the extreme conditioning program training present a high level of self-determination, and the subjects may experience a greater sense of satisfaction and pleasure (Dominski et al., 2020). Therefore, it is pertinent to examine the motivational characteristics of individuals who engage in this type of training modality.

Regarding functional training, previous studies showed high public acceptance of this type of modality due to the facilitation characteristics of some barriers to the beginning and maintenance of physical exercise, such as lack of motivation and time for practice (Trost et al., 2002). As in extreme conditioning programs, functional training classes involve relevant contextual factors during exercise that can help increase and maintain adherence to training program, such as using music as a reference for participants to control exercise intensity through rhythm (Karageorghis and Priest, 2012), and a group environment which can represent a positive modulator to the motivation of the practice of this modality.

Regarding lack of time, both extreme conditioning programs and functional training use a time-efficient approach, which can help to increase the readiness to engage in physical activities, with high-intensity interval training representing a relevant choice for the continuous training method, as it involves significantly less time (Wilke et al., 2019).

Therefore, the result of this study is consistent with previous research, in that the pursuit of pleasure for the practice of physical exercise leads to increased persistence, positive psychological feelings, and reduced stress, leading the practitioner to a greater propensity to feel energized, confident, and satisfied with the activity itself (Fisher et al., 2016). For profile 2, medium and high levels of autonomy were verified, indicating more self-determined motivation profiles, with evidence showing that people with this profile tend to have greater personal perception associated with regular physical exercise, with greater satisfaction, effort, persistence, and defined intention to remain involved with the chosen modality for a longer time (Vlachopoulos et al., 2000). This occurs once the practitioner exhibits a more internally regulated behavior, in which the individual considers it important and perceives the value of the benefits provided by the performed activity (Wilson et al., 2003).

Finally, profile 3 shows that the participants of this study who practice Pilates seek “stress control” as an objective. The Pilates modality provides a wide and complete coordination between body, mind, and spirit, being based on principles that allow to increase attention and motivation, and improve cognitive functions, minimizing body stress (Ungaro and Sadur, 2002). In the present study, it was found that individuals seek Pilates practice aiming more than improving their health in general, but also optimizing results such as gaining strength, flexibility, improved posture, motor coordination, breathing, and stress control. These outcomes corroborate with findings of the study by Memmedova (2015). The authors demonstrated that Pilates leads to a positive mood modulation and reduces the tension and anxiety suffered by the individual in daily life. Corroborating these results, Vancini et al. (2017) found positive effects of Pilates training on quality of life, depression, and trait anxiety scores in overweight and obese individuals.

In an investigation conducted by Ahmadi and Mehravar (2019), the effect of 60 min of Pilates, three times a week, for 8 weeks, in controlling stress and cortisol levels in 22 sedentary women between 25 and 40 years was investigated. In addition to the improvement in body composition, resting heart rate, and blood pressure, cortisol levels and perceived stress showed lower values after the intervention. It is known that cortisol hypersecretion is associated with mental disorders, with some studies showing lower stress induction and lower cortisol concentration in physically trained individuals compared to untrained individuals (Traustadóttir et al., 2005). Thus, physical exercises with the characteristics of Pilates, in which the practitioner observes increased body awareness and conscious use of breath, can act as a protection against exaggerated or sustained stress responses, promoting better stress management, and relaxation (Adams et al., 2012).

Although the present study has evaluated associations between motivational factors, personal characteristics regarding the objective for the practice and modalities of physical exercise, there is still diversity and differences between the reasons and barriers that determine adherence to physical exercise, and the results of this study are essentially descriptive characteristics to elucidate possible associations between the variables of interest. Thus, cause and effect inferences should not be determined with the results found providing managerial implications, by enabling health professionals (especially physical education professional) to better visualize certain profiles of physical exercise practitioners, who are influenced by qualitative and perceptual aspects.

In addition, it is important to consider some limitations of the present findings. No intervention was induced on the sample and due to the social distance resulting from the COVID-19 pandemic strengthened the already emergent process of virtual connections between people, bringing implications also for conducting this research. For this reason, platforms that allow online interviews were considered to collect data. The other limitation was about sample size (recruitment) in which fitness centers/gyms work further subject to a limitation of 50 persons or less or in some instances, exercise was prohibited during the lockdown.

Forthcoming experimental studies are needed to address the motivation levels and goals for the practice of physical exercise in different modalities considering groups with or without association between motivation levels (low, medium, and high levels of autonomy), modalities practiced (extreme fitness program, strength training, fight training, Pilates, and functional training), and goals (sociability, competitiveness, stress control, health, aesthetics, and pleasure) concerning the practice of physical exercise on gym clients exercise adherence considering attendance.

Therefore, a possibility of handling qualitative data through a multiple correspondence analysis may display a perceptual map with the diversity of behavioral factors and how they relate to the desires and needs of those who seek physical exercise. Other areas, such as marketing, utilize the treatment of these categorical data as a simple and viable alternative to stratify the market into niches with homogeneous and representative behavior profiles, so that sales strategies are more effective. To promote and encourage the regular practice of physical exercise, this strategy could be used to take actions that increase the public’s intention to start or continue to be involved in the regular practice of physical exercise.

Three profiles were observed by associating levels of motivation and goals for the practice of physical exercise in five different modalities evaluated. Participants in the strength training and fight training modalities seek these modalities with the objective of “health” and “aesthetics,” demonstrating low autonomy in relation to the behavior for the practice of physical exercise; the participants of the extreme conditioning program and functional training modalities have as objective the search for “pleasure” with the practice of physical exercise, demonstrating medium and high autonomy for such practice. Finally, Pilates practitioners have the objective of “stress control.”

## Data Availability Statement

The raw data supporting the conclusions of this article will be made available by the authors, without undue reservation.

## Ethics Statement

The studies involving human participants were reviewed and approved by Research Ethics Committee of the Universidade Estácio de Sá (CAAE 34538220.2.0000.5284). The patients/participants provided their written informed consent to participate in this study.

## Author Contributions

This study was conducted by JB, GO, and JR. JR, CL, and RS proposed the idea, performed the analyses, and helped JB and GO to draft the manuscript. EA and MB supervised and guided the writing of the manuscript. All authors read and approved the final manuscript.

## Funding

This study was supported by Programa Institucional de Bolsas de Iniciação Científica (PIBIC) – Edital de Concurso para Seleção de Projetos de Iniciação Científica e de Cotas de Bolsas 08/2020 – 07/2021 of Centro Universitário Estacio De Belo Horizonte. This study was financed in part by the Coordenação de Aperfeiçoamento de Pessoal de Nível Superior - Brasil (CAPES) - Finance Code 001. CABL is a productivity fellowship at the Conselho Nacional de Desenvolvimento Científico e Tecnológico (CNPq, Brazil).

## Conflict of Interest

The authors declare that the research was conducted in the absence of any commercial or financial relationships that could be construed as a potential conflict of interest.

## Publisher’s Note

All claims expressed in this article are solely those of the authors and do not necessarily represent those of their affiliated organizations, or those of the publisher, the editors and the reviewers. Any product that may be evaluated in this article, or claim that may be made by its manufacturer, is not guaranteed or endorsed by the publisher.
